# Evolution characteristics and multi-scenario prediction of habitat quality in Yulin City based on PLUS and InVEST models

**DOI:** 10.1038/s41598-024-62637-4

**Published:** 2024-05-24

**Authors:** Shifeng Li, Zenglin Hong, Xuping Xue, Xiaofeng Zheng, Shaoshao Du, Xiaofeng Liu

**Affiliations:** 1 School of Land Engineering, Chang’an University, Xi’an, 710054 China; 2 Shaanxi Institute of Geological Survey, Xi’an, 710054 China; 3 Shaanxi Hydrogeology Engineering Geology and Environment Geology Survey Center, Xi’an, 710054 China

**Keywords:** Land use evolution, Plus model, Invest model, Habitat quality, Multi-scenario prediction, Ecology, Environmental social sciences

## Abstract

As a major energy city in China, Yulin City has faced huge challenges to the ecological environment with its rapid economic development and rapid urbanization. Therefore, it is of great significance to study the impact of land use changes on habitat quality. Based on three periods of land use data in Yulin City in 1995, 2005 and 2015, the PLUS model was used to simulate the land use changes in 2015. The measured kappa coefficient was 0.8859, which met the simulation accuracy requirements. By setting development zone boundaries and adjusting parameters, three progressive scenarios are designed to predict the spatial distribution of land use in Yulin City in 2035. The InVEST model was used to analyze the spatiotemporal evolution of Yulin City’s habitat quality in the past 20 years and evaluate the distribution of Yulin City’s habitat quality under three scenarios after 20 years. The results are as follows: (1) During the study period, construction land in Yulin City expanded rapidly, with an area increase of 380.87 km^2^ in 20 years, and ecological land gradually shrank. (2) The land use simulation results of Yulin City under various scenarios in 2035 show that future land use changes in Yulin City will mainly be concentrated in the central and western regions. (3) During the study period, the habitat quality of Yulin City was at a medium level and the overall habitat quality showed a downward trend. Spatially, the degree of habitat quality degradation in Yulin City showed a characteristic of gradually decreasing from West to East. (4) By 2035, under the scenario of suitable urban economic development, Yulin City’s habitat quality has been improved to a certain extent, which not only protects ecological security but also meets the demand for construction land for urban development. The results of this study help the government better understand the evolution of land use and habitat quality in Yulin City in the past 20 years, and provide theoretical support and reference for the formulation of Yulin City’s ecological environment protection policies and the implementation of ecological protection work under the current land spatial planning.

## Introduction

Habitat quality refers to the ability of the natural environment to sustainably provide individuals or populations with the necessary conditions for survival and development within a certain region. It is an important factor in maintaining regional sustainable development^1,2^. With the advancement of urbanization, population growth and the continuous expansion of construction land have led to the loss of ecological service areas such as forests, grasslands, and wetlands, changing the habitat distribution pattern, resulting in the continuous reduction and fragmentation of habitat patch landscape connectivity, affecting the energy flow and material circulation between different habitat patches^3–5^. Therefore, analyzing land use evolution characteristics, simulating future urban land use changes, and studying the impact of land use changes on habitat quality is of great significance for regional formulation of land resource optimization and ecological protection policies, and for solving the coordinated development of humans and ecology^6^.

At present, most land use change prediction models are based on meta-cellular automata, such as CA-Markov model^7–9^, CA-MAS model^10^, CLUE-S model^11–13^ and FLUS model^14–16^. However, the Markov model has an absolute advantage in predicting the amount of land in the long term in the future, but it cannot predict changes in the spatial distribution pattern of land use^17^. The CA-MAS model is difficult to reflect the impact of socioeconomic conditions and other factors on urban land use patterns^18^. The CLUE-S model ignores the possibility of non-dominant land class conversion in its application, requiring the use of separate mathematical models for non-spatial modules^19^. The FLUS model can simulate under the joint action of multiple land use types, and can effectively handle the complexity and uncertainty of land conversion under the joint influence of natural and social factors. However, the simulation accuracy is slightly lower than the PLUS model under the same parameter settings^20^. The land expansion analysis method (LEAS) and CA based on the multiple random seed analysis method (CARS) were introduced in the patch generation land use change simulation (PLUS) model to solve these problems. It combines the advantages of high accuracy and high speed at the same time, allowing it to simulate the complex evolution of various land types^21^. By adjusting the model parameters, the needs of different scenario simulations in different regions and under different strategies can be better met. The model performs well in applications at different spatial scales, such as counties^22^, cities^23,24^, and provinces^25^.

Land use changes affect the distribution pattern and structure of habitat, thereby affecting regional habitat quality. At present, the habitat quality evaluation models mainly include ARIES (Artificial intelligence for ecosystem services) model^26^, SoLVES (Social values for ecosystem services) model^27^, HSI (Habitat suitability index) model^28^ and InVEST model^29^. Among them, the InVEST model has the characteristics of simple operation, small data requirements, and strong spatial expression ability, and is widely used in habitat quality assessment^30^. It has been successfully used in urban agglomerations^31,32^, provinces^33,34^, and cities^35,36^ research on habitat quality at different scales.

As a major energy city in China, Yulin City is also a key city in the Yellow river key ecological zone, a national key ecological functional area. With the continuous exploitation of energy resources in Yulin City, rapid economic development, and rapid urbanization, the ecological environment has been faced with huge challenges. Therefore, this paper uses the land use data of Yulin City in 1995, 2005 and 2015 to simulate and analyze the land use changes and habitat quality of Yulin City in 2035 under multiple progressive scenarios. In addition, this article also discusses the land use evolution characteristics of Yulin City in the past 20 years and the relationship between habitat quality and urban expansion. The research results can provide a reference basis for Yulin City to formulate land resource allocation and ecological environment protection policies.

## Materials and methods

### Overview of the research area

**Figure 1:**
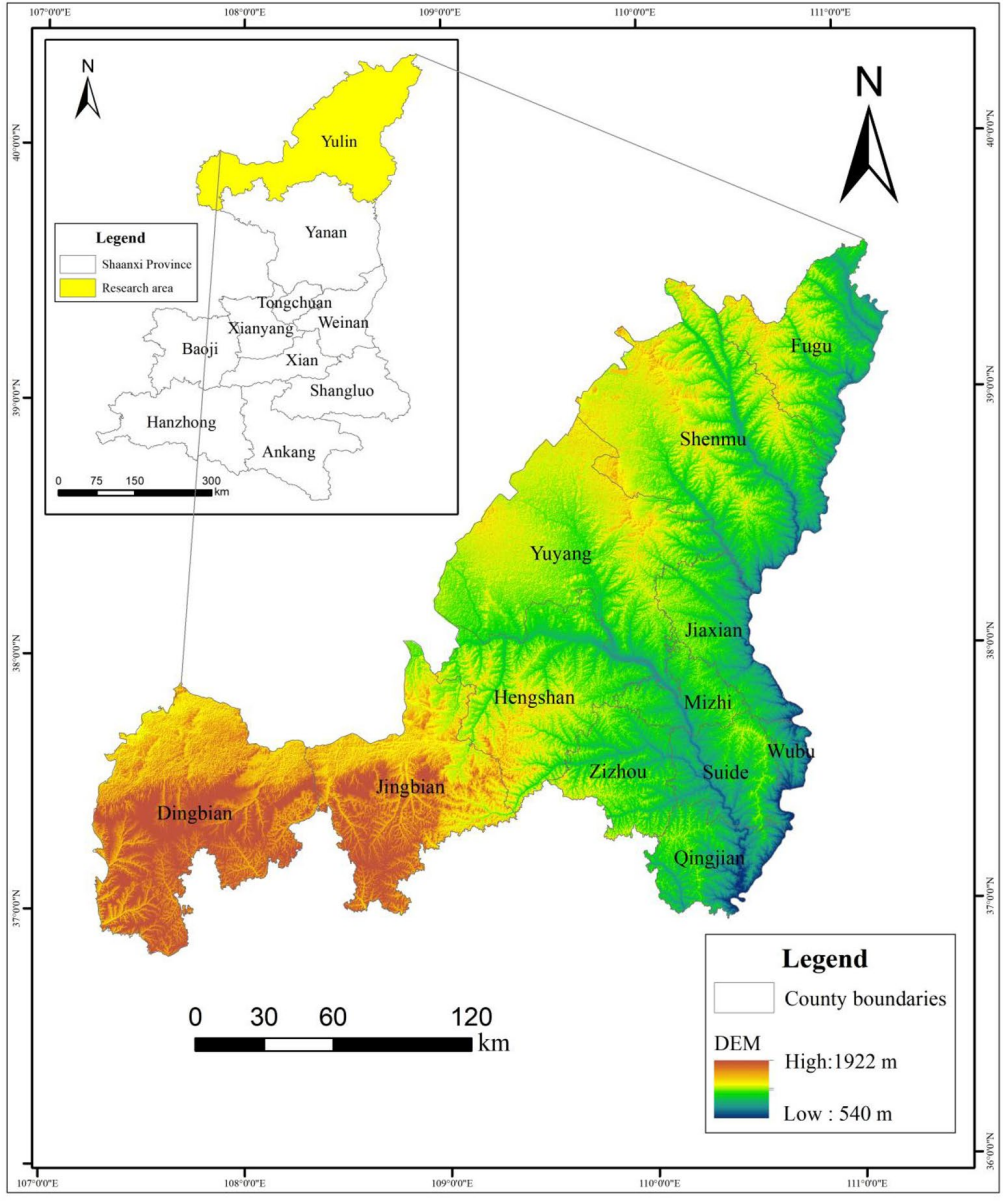
Overview map of the study area. The maps were created using the free and open source ArcGIS, Version 10.5 (https://www1.jjjzfx.cn/gis-jb61d/).

Yulin City (36°57′ ~ 39°35′N, 107°28′ ~ 111°15′E) is located in the northernmost part of Shaanxi Province, It faces Shanxi on the east by the Yellow River, Ningxia and Gansu on the west, Inner Mongolia on the north, and Yan’an City on the South. The city has jurisdiction over 1 city, 2 districts and 9 counties, with a total area of 42,920.2 square kilometers and an altitude of between 1000 and 1350 m. The city’s landforms are divided into three categories: windy sand and grassland areas, loess hilly and ravine areas, and beam-shaped low hills and hilly areas (Fig. 1). Yulin City belongs to the temperate arid and semi-arid continental monsoon climate zone, with four distinct seasons, a dry climate and less precipitation. As of the end of 2022, the city’s permanent population is 3.6161 million, including 2.2528 million urban residents. The urbanization rate of the city’s permanent population has reached 62.3%, and it is in the process of rapid urbanization.

### Data sources and processing

**Figure 2:**
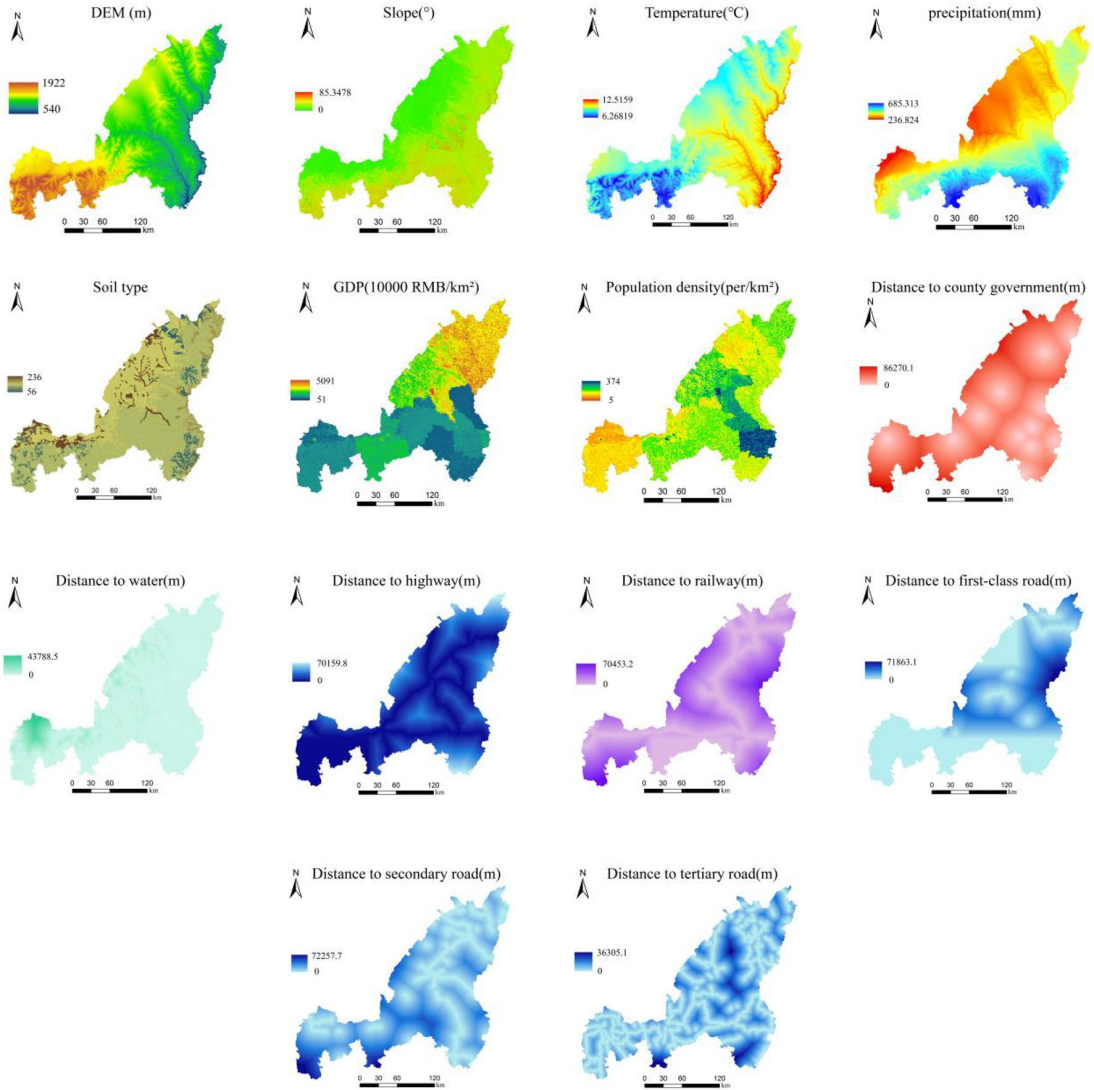
Main influencing factors of land use change. The maps were created using the free and open source ArcGIS, Version 10.5 (https://www1.jjjzfx.cn/gis-jb61d/).

The three-phase land use data required for this study comes from the resource and environmental science data center of the chinese academy of sciences (https://www.resdc.cn/Datalist1.aspx?FieldTyepID=1,3), with a resolution of 30 × 30 m. The land use data of each period is reclassified into six categories: cultivated land, forest land, grassland, water area, construction land and unused land. Land use change is closely related to the natural environment, traffic location, socioeconomic and other factors. Based on relevant literature research^37,38^ and considering the availability of data, this paper finally selected the main spatial variables that affect land use change to include natural factors (elevation, slope, soil type, temperature and precipitation), transportation location factors (distance from town centers, rivers, railways, highways, primary roads, secondary roads and tertiary roads) and socioeconomic factors (population and GDP) (Fig. 2). Among them, the DEM data comes from the geospatial data cloud (https://www.gscloud.cn/), the slope is extracted from the DEM data, and the soil type, temperature, precipitation, population and GDP data come from the resource and environment data sharing center of the chinese academy of sciences (https://www.resdc.cn/). Data such as water systems, roads and administrative centers come from the national geographic information resources directory service system (https://www.webmap.cn/main.do?method=index), and ArcMap spatial analysis tools are used to perform Euclidean distance analysis on water systems, roads and administrative centers. The resolution of all raster data is unified to 30 × 30 m, the coordinate system projection is CGCS2000_3_Degree_GK_Zone_36, and the raster range remains consistent. The detailed parameters are shown in Table 1.

**Table 1:** Basic and driving factor data of research.

Type	Name	Data time	Spatial resolution	Data source	Data processing and use
Basic data	Yulin city land use data	1995, 2005, 2015	30 m	Resource and environmental science data center of the Chinese academy of sciences	ArcGIS was used to reclassify the land use data of the third period into six categories. Data used to simulate and predict future land use changes
	Administrative boundaries of Yulin City	2015	–		
Natural drivers	DEM	2015	30 m	Geospatial Data Cloud	
	Slope	2015	30 m		
	Soil type	2015	30 m	Resource and environmental science Data center of the Chinese academy of sciences	
	Temperature	1995, 2005, 2015	30 m		
	Precipitation	1995, 2005, 2015	30 m		
Socio-economic drivers	Yulin GDP spatial distribution grid data	1995, 2005, 2015	1 km		Use ArcGIS raster data conversion tools to convert GDP and population data to 30-m resolution
	Yulin Population Spatial Grid DistributionData	1995, 2005, 2015	1 km		
Traffic location driveers	Distance to administrative centers	2015	–	National geographic information resources directory service system	ArcMap10.5 was used to perform Euclidean distance analysis on the data, which was used by the PLUS model to predict future land use changes
	Distance to river				
	Distance to railway				
	Distance to highway				
	Distance from primary road				
	Distance from secondary road				
	Distance from tertiary road				

### Research methods

#### Research framework

**Figure 3:**
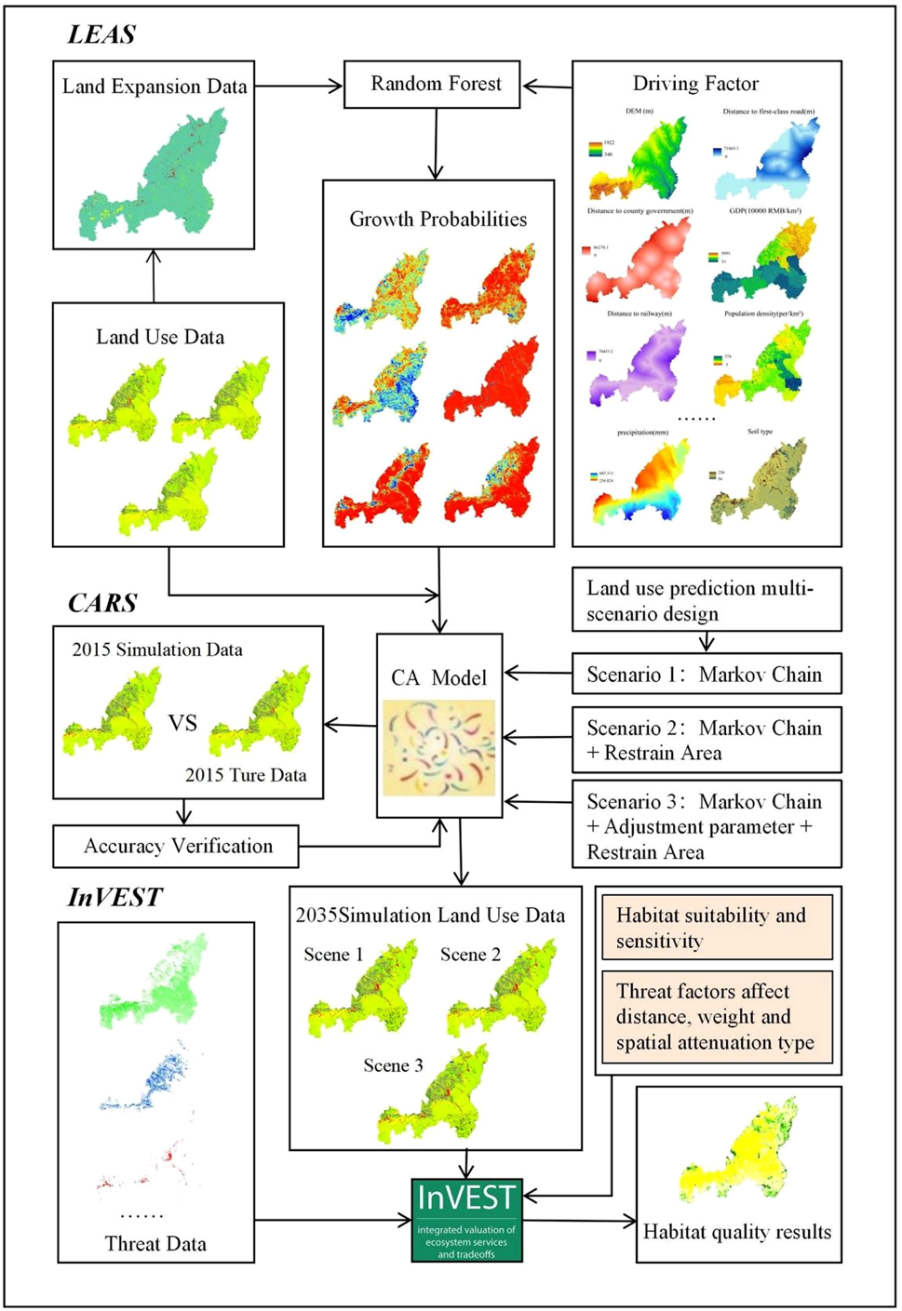
Technology roadmap. The maps were created using the free and open source ArcGIS, Version 10.5 (https://www1.jjjzfx.cn/gis-jb61d/).

Based on the land use data of Yulin City from 1995 to 2015, the PLUS model was used to predict the spatial distribution of land use in Yulin City in 2035 under three scenarios, and the InVEST model was used to analyze the spatiotemporal evolution of Yulin City’s habitat quality from 1995 to 2015. And evaluate the habitat quality of Yulin City in 2035 under different development scenarios. The specific method and process are shown in Fig. 3.

#### PLUS model

The PLUS model (patch-generating land use simulation) integrates the land expansion analysis strategy (LEAS) module and the CA model (CARS) based on multi-class random patch seeds to explore the driving factors of land expansion and predict future land use landscapes. Patch-level evolution. First, the LEAS module is used to evaluate the driving forces and spatial characteristics of various types of land use expansion between the two periods of land use data, extract the parts of various types of land use expansion between the two periods of land use changes, use the random forest algorithm to sample the land expansion, and calculate each The development probability of land types^39^, its mathematical expression is as follows:

1 $$ P_{i,k}^{d} \left( x \right) = \frac{{\mathop \sum \nolimits_{n = 1}^{M} I = \left[ {h_{n} \left( x \right) = d} \right]}}{M}, $$

where ${P}_{i,k}^{d}(x)$ is the growth probability of land use type k at cell i, d either 0 or 1. If d = 1, then k landuse type have replaced all other land use types; If d = 0, then land use types have replaced all other land use types except k type. x is a vector composed of several driving factors; the function I is the indicator function of the decision tree set; hn(x) is the prediction type of the *n*th decision tree of the vector x; M is the total amount of the decision tree.

Then use the CARS module to simulate and predict land use. CARS is a scenario-driven land use simulation model. During the simulation process, the model combines random seed generation and threshold decreasing mechanisms to generate land use patches under the constraints of adaptation coefficient, neighborhood effect, development probability and transition matrix^21^. Neighborhood weight is an important indicator in land use simulation, with a value ranging from 0 to 1. The larger the value, the stronger the expansion ability of the land type. It can be calculated based on the proportion of the expansion area of each land use type. The land use transfer matrix indicates whether different land types can be converted to each other. 0 means that conversion to other land types cannot occur, and 1 means that conversion to other land types can occur.

#### InVEST model

The habitat quality module of the InVEST model is based on land use type data and evaluates habitat quality based on factors such as the relative sensitivity of each habitat type to each threat factor, threat factor data, maximum impact distance of threat sources, and impact weight. The habitat quality index ranges from 0 to 1. The larger the value, the better the habitat quality. The calculation formula is:

2 $$ Q_{xj} = H_{j} \left( {1 - \frac{{D_{{xj^{z} }} }}{{D_{{xj^{z} }} + K^{z} }}} \right), $$

Where the habitat quality index is Q_xj_; H_j_ is the suitability of the habitat for land type j, and the range of values is [0, 1], with 1 being the highest habitat quality; The habitat degradation level for raster cell x in land type j is D_xj_; K is a half-saturation constant with a default value of 0.5, usually half of the maximum degradation degree; z is a normalization constant with a default value of 2.5; the following formula is used to calculate Dxj:

3 $$ D_{xj} = \mathop \sum \limits_{r = 1}^{R} \mathop \sum \limits_{y = 1}^{{Y_{r} }} \left( {\frac{{w_{r} }}{{\mathop \sum \nolimits_{r = 1}^{R} w_{r} }}} \right)r_{y} i_{rxy} \beta_{x} S_{jr} , $$

4 $$ i_{rxy} = 1 - \left( {\frac{{d_{xy} }}{{d_{r max} }}} \right) \left( {{\text{Linear}}} \right), $$

5 $$ i_{rxy} = exp\left( {\frac{{ - 2.99d_{xy} }}{{d_{r max} }}} \right) \left( {{\text{Exponential}}} \right), $$

Where Y_r_ is the number of stress factor grid cells, y represents all of the stress factor’s grid cells, and R is the number of stress factors. The weight of the stress factor r is W_r_ . r_y_ represents the value of the stress variables for the raster y; i_rxy_ is the stress level of the stress factor ry of raster y to raster x; β_x_ is the accessibility of stress factor to raster x; S_jr_ is the habitat type j’s sensitivity to the stress factor r with a value range of (0,1); The linear distance between the grids x and y is denoted by d_xy_ ; and the maximum stress distance for the stress factor r is denoted by d_rmax_.

Based on relevant literature^40–42^ and other scholars’ research and combined with the actual situation of Yulin City, cultivated land, urban land, rural residential areas, other construction land and unused land were selected as threat factors, and the threat factor weight and maximum influence distance (Table 2) and the habitat suitability and sensitivity to threat factors of various species are set according to actual conditions (Table 3).

**Table 2:** Stress factors affect distance, weight and spatial attenuation type.

Threat factor	Maximum influence distance/km	Weight	Spatial decay type
Cultivated land	3	0.5	Linear
Urban land	10	0.9	Exponential
Rural settlements	5	0.7	Exponential
Other construction land	8	0.8	Exponential
Unutilized land	8	0.4	Linear

**Table 3:** Habitat suitability of each species and sensitivity to different threat factors.

Land type	Habitat suitability	Cultivated land	Urban land	Rural settlements	Other construction land	Unutilized land
Cultivated land	0.4	0.3	0.5	0.35	0.4	0.4
Forestland	1	0.6	0.85	0.65	0.6	0.55
Grassland	0.75	0.55	0.7	0.5	0.55	0.7
Water body	1	0.65	0.8	0.68	0.7	0.3
Construction land	0	0	0	0	0	0
Unutilized land	0.1	0.2	0.1	0.1	0.1	0.1

#### Multi-scenario design of land use prediction

This study selected three scenarios of natural development, ecological protection and urban economic appropriate development to predict and simulate the land use of Yulin City in 2035.

- (1) Natural development scenario: Without considering human factors, prediction and simulation are carried out according to the historical urban expansion trend. The land use transition probability is the same as the transition probability matrix from 2005 to 2015.
- (2) Ecological protection scenario: According to water and woodland as the key objects of ecological protection, water and woodland are used as constraints and will not be transferred to other land types.
- (3) Suitable development scenario of urban economy: According to statistics, Yulin City’s urbanization rate increased from 55.51% in 2015 to 61.6% in 2020 in the 13th 5 Year Plan, an increase of 6.09%. In the 14th 5 Year Plan, it is expected that the urbanization rate of Yulin City will reach 67% and a new urban system integrated with the city will be built. Therefore, the probability of transferring other land types to construction land will increase by 20%. In order to maintain the safety of ecological land, water areas and forest lands are set up so as not to be transferred to other land types.

Results and analysis

### Land use change characteristics and predictions

#### Analysis of land use characteristics from 1995 to 2015

The land use changes in Yulin City from 1995 to 2015 were characterized by a sharp increase in construction land, a continuous increase in forest land, and a sharp decrease in cultivated land (Table 4). The main manifestation is that the construction land area in Yulin City has increased by 380.87 km^2^ in 20 years, accounting for approximately 245% of the original construction land area; From 1995 to 2005, the change rate of construction land area was 4.18 km^2^/a, and from 2005 to 2015, the change rate increased to 33.91 km^2^/a. The forestland area increased by 797.69 km^2^, accounting for about 50% of the original forestland area. From 1995 to 2005, the forestland area change rate was 73.77 km^2^/a, and from 2005 to 2015, the change rate dropped to 6 km^2^/a. Unused land increased by 525.51 km^2^, accounting for approximately 13% of the original unused land area, and its area change rate was 26.27 km^2^/a. In addition, the area of other land types has decreased within 20 years, of which the cultivated land area has decreased by 1368.23 km^2^, accounting for about 7.9% of the original cultivated land area. From 1995 to 2005, the change rate of cultivated land area was 94.26 km^2^/a, from 2005 to in 2015, the change rate dropped to 42.57 km^2^/a. The grassland area decreased by 283.36 km^2^ (the change rate is 14.17 km^2^/a). The grassland area decreased by 491.79 km^2^ in the first 10 years, and the grassland area increased by 208.43 km^2^ in the next 10 years; the water area decreased by 52.49 km^2^ (the change rate is 2.62 km^2^/a).

**Table 4:** Area changes of land use types in the three periods from 1995 to 2015 (unit: km^2^).

Land type	Cultivated land	Forestland	Grassland	Water body	Construction land	Unutilized land
The year 1995	17265.63	1583.65	19459.49	550.57	155.37	3955.21
The year 2005	16323.06	2321.34	18967.71	526.31	197.17	4634.32
The year 2015	15897.39	2381.35	19176.13	498.07	536.24	4480.73

**Figure 4:**
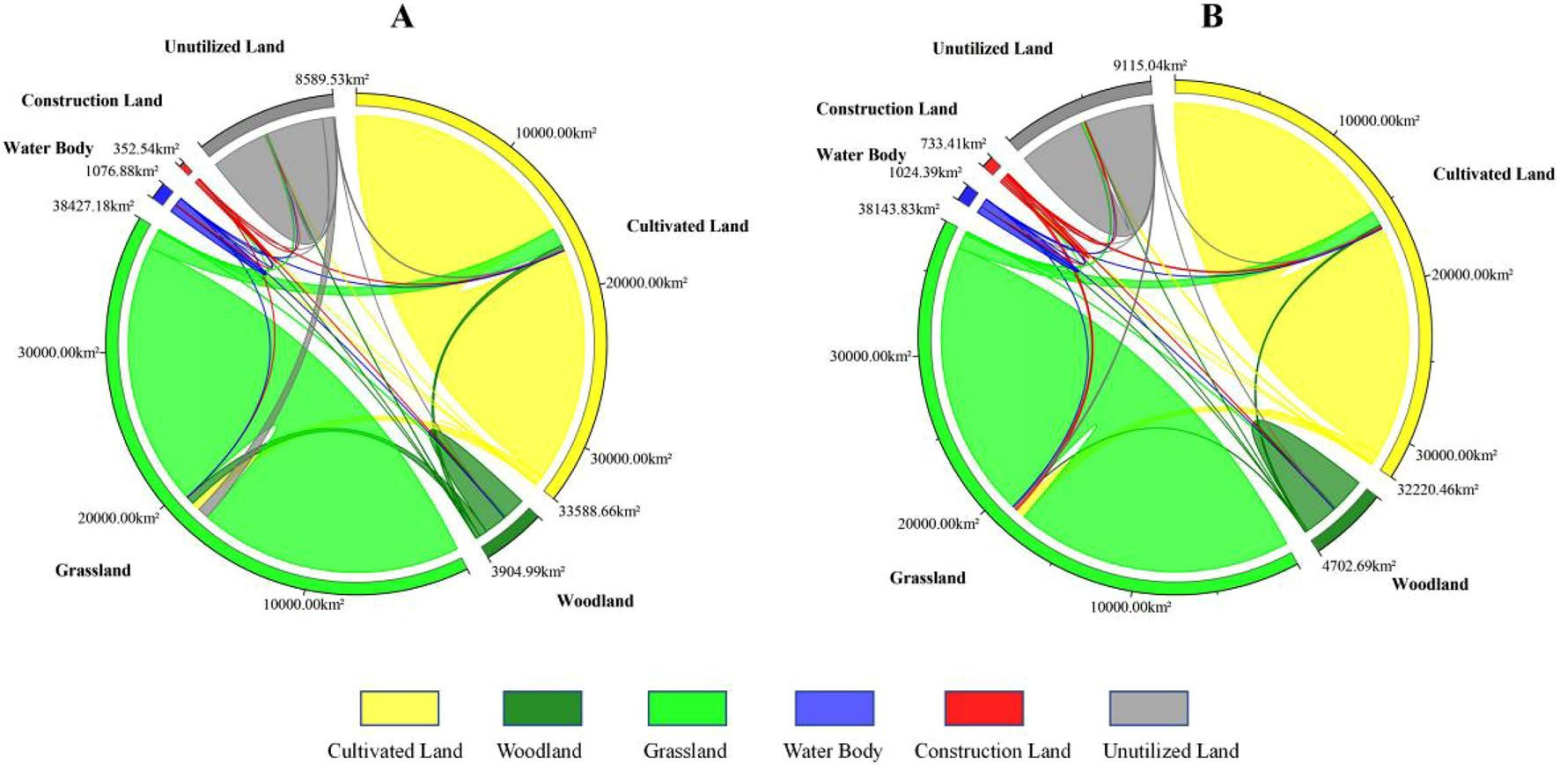
Land use conversion ratio chord chart for the years (**A**) 1995–2005 and (**B**) 2005–2015.

According to the land use conversion matrix in these two periods, it can be seen that the rapid increase in forestland, construction land and unused land in Yulin City from 1995 to 2005 was mainly due to the transfer of cultivated land and grassland, and its cultivated land transfer area (942.56 km^2^) accounted for 32.3% of the total transferred area (2917.21 km^2^), and the grassland transferred area (491.78 km^2^) accounted for 16.9% of the total transferred area (2917.21 km^2^) (Fig. 4A). The rapid growth of construction land area in Yulin City from 2005 to 2015 was mainly due to the transfer of cultivated land and grassland. Its transferred area (339.06 km^2^) accounted for 27.9% of the total transferred area (1215 km^2^). The transfer of cultivated land and unused land is the main reason for the rapid growth of grassland area, and its transferred area (208.43 km^2^) accounts for 17.2% of the total transferred area (1215 km^2^) (Fig. 4B).

**Figure 5:**
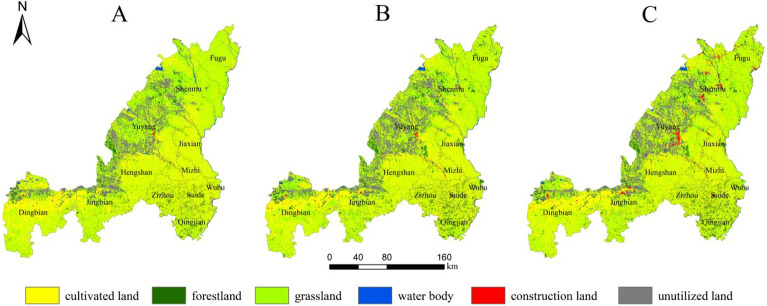
Land use situation of Yulin in (**A**) 1995, (**B**) 2005, and (**C**) 2015. The maps were created using the free and open source ArcGIS, Version 10.5 (https://www1.jjjzfx.cn/gis-jb61d/).

Spatially, the main evolutionary characteristic of Yulin City is that with the continuous expansion of construction land, the ecological space continues to decrease (Fig. 5A–C). During the study period, the urban spatial expansion of Yulin was mainly external expansion, such as Fugu County, Shenmu City, Yuyang District, Jingbian County, Dingbian County and Suide County, by encroaching on external ecological spaces such as cultivated land, grassland and water bodies. Expansion to meet the requirements of urban development during the study period.

#### Multi-scenario analysis of land use simulation

Based on the land use data in 1995, 2005 and 2015, the PLUS model was used to predict the spatial distribution of land use in Yulin City in 2035 under three scenarios. First, the LEAS module in the PLUS model is used to calculate the growth probabilities of six land use types based on current land use data and 14 natural, traffic location and socioeconomic driving factors. Then the CARS module was used to simulate the land use situation in 2015, and compared with the actual land use situation in Yulin City in 2015, the Kappa coefficient was 0.8859, and the overall accuracy was 0.9259. It shows that the simulation results have high spatial consistency and meet the simulation accuracy requirements of this study. Therefore, the real land use data of Yulin City in 2015 was used to predict the land use situation of Yulin City in 2035.

**Figure 6:**
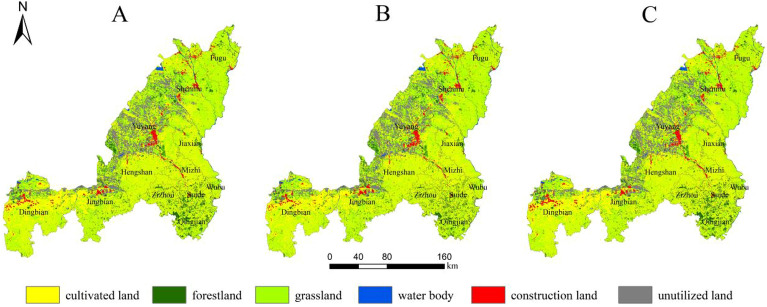
Land use situation of Yulin city in 2035 under the scenarios of natural development (**A**), ecological protection (**B**) and suitable development of urban economy (**C**).The maps were created using the free and open source ArcGIS, Version 10.5 (https://www1.jjjzfx.cn/gis-jb61d/).

**Figure 7:**
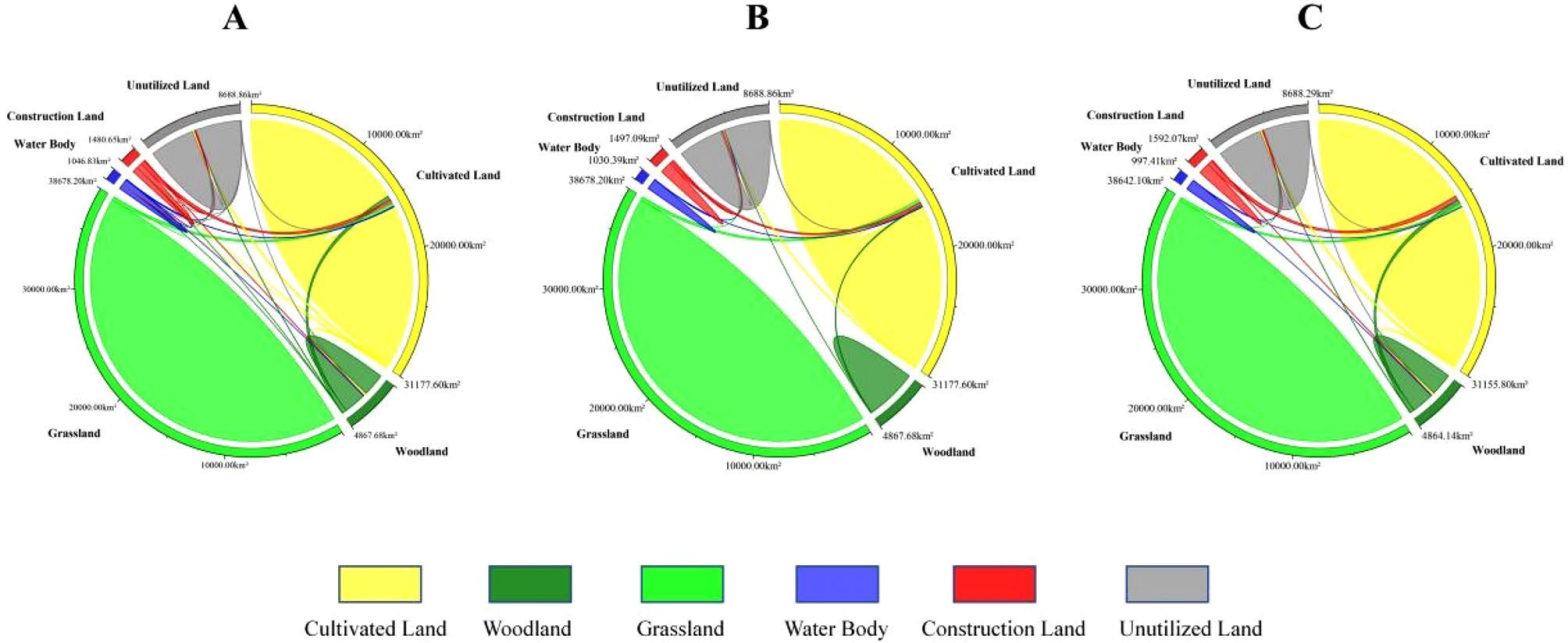
The chord diagram of land use conversion in 2035 Yulin under the scenarios of natural development (**A**), ecological protection (**B**) and suitable development of urban economy (**C**).

By simulating the land use situation in Yulin City in 2035, it can be seen that the land use types in many areas have changed (Table 5). In the natural development scenario, urban development continues historical trends, and each changing area changes land use types through external expansion. Among them, construction land in the central and western regions represented by Dingbian County, Jingbian County, Hengshan District and Yuyang District has expanded rapidly (Fig. 6A). Under this scenario, the construction land area increases by 408.18 km^2^, of which the cultivated land transfer area is 314.32 km^2^, accounting for 77% of the increased construction land area; The ecological land area of forestland and grassland decreased by 1.08 and 27.06 km^2^ respectively (Fig. 7A). In this case, since there are no restrictions on land conversion, woodland and grassland in the planned ecological reserve are encroached by construction land, indicating that urban expansion under natural scenarios will cause damage to the urban ecosystem.

**Table 5:** Area changes of each land use type under three scenarios in 2035 (unit: km^2^).

Land type	Cultivated land	Forestland	Grassland	Water body	Construction land	Unutilized land
Natural development scenario	15459.69	2380.27	19149.07	548.75	944.42	4487.71
Ecological protection scenario	15280.21	2486.33	19502.06	532.32	960.85	4208.13
Scenario of suitable urban economic development	15258.41	2482.79	19465.97	499.34	1055.84	4207.56

Under the ecological protection scenario, since clear boundary restrictions are set on urban expansion, forest land and water areas are not transferred to other land types, thus protecting the ecological space in the restricted area (Fig. 6B). Under this scenario, in 2035, Yulin City’s woodland, grassland and water areas will increase by 104.98, 325.94, and 34.25 km^2^ respectively; the construction land area will increase by 424.62 km^2^, and the cultivated land and unused land areas will decrease by 617.18 and 272.61 km^2^ respectively (Fig. 7B), This shows that ecological protection priority plays an important role in the optimization and maintenance of urban ecosystems.

Under the scenario of suitable urban economic development, clear boundary restrictions are set for the city and the requirements of urban planning are met (Fig. 6C). Under this scenario, the construction land area in Yulin City increased by 519.60 km^2^ in 2035, and the areas of forestland, grassland and water increased by 101.44, 289.84 and 1.26 km^2^ respectively; the area of cultivated land and unused land decreased by 638.98 and 273.161 km^2^ respectively (Fig. 7C). This shows that in this scenario not only the ecological land space is protected, but also the demand for construction land for future urban development is met.

### Comparative analysis and multi-scenario simulation of spatiotemporal evolution of habitat quality

#### Habitat quality evolution characteristics of Yulin City from 1995 to 2015

Land use changes affect the distribution pattern and structure of habitats, thereby leading to changes in regional habitat quality. On the basis of analyzing the evolution of land use in Yulin City, the habitat quality of Yulin City in different periods was evaluated, and the changing characteristics and response relationship between land use and regional habitat quality were further explored. By importing the existing three-phase land use data, threat factor data and factor sensitivity data into the InVEST model, the habitat quality data of Yulin City in 1995, 2005 and 2015 were obtained. The habitat quality value range is (0–1). The larger the value, the better the habitat quality, the more comprehensive the ecosystem services, and the higher the ecological value. In order to better visualize the evolution process of habitat quality, the natural break point classification method in ArcGIS was used to classify the habitat quality results into four categories: poor (0–0.25), medium (0.25–0.5), good (0.5–0.75) and excellent (0.75–1).

**Figure 8:**
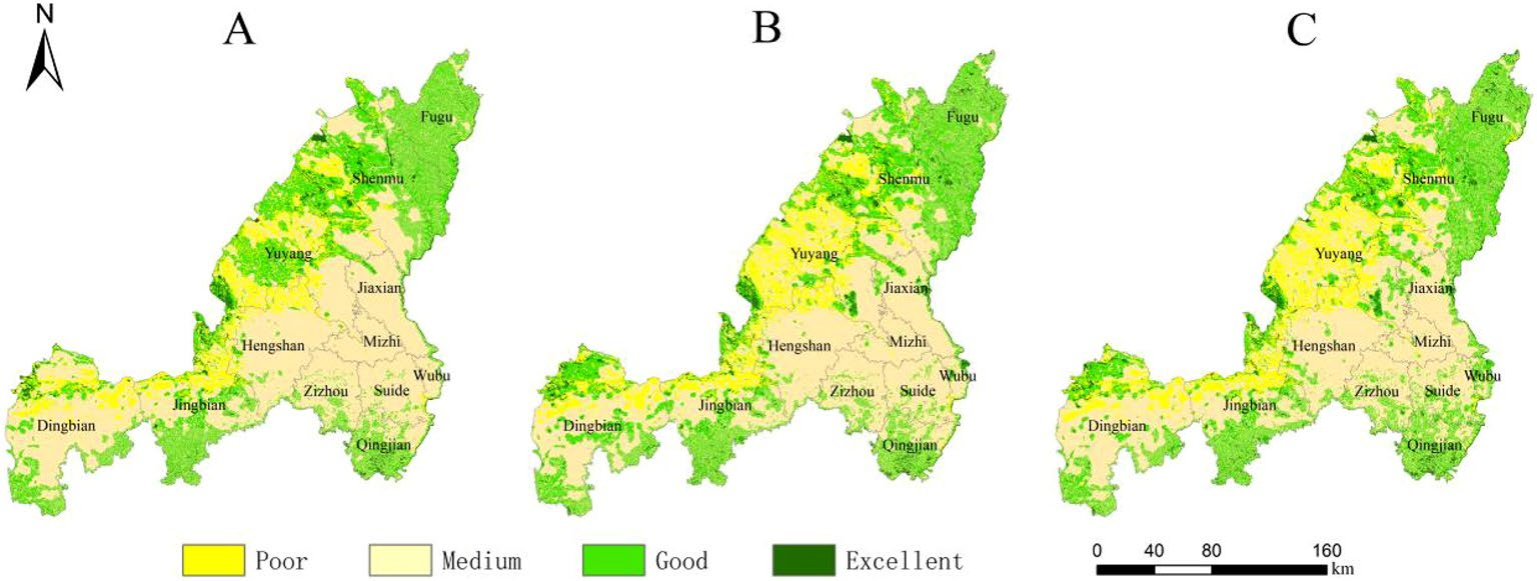
Spatial distribution of Yulin’s habitat quality in 1995(**A**), 2005 (**B**) and 2015(**C**). The maps were created using the free and open source ArcGIS, Version 10.5 (https://www1.jjjzfx.cn/gis-jb61d/).

From the perspective of classification levels, the habitat quality of Yulin City has been at a medium level since 1995, and the overall habitat quality has shown a downward trend. In the 20 years of the study, the area of medium habitat quality decreased by 1506.93 km^2^, and the area of poor habitat quality increased by 968.94 km^2^. From the perspective of spatial distribution, degraded habitat quality is mainly located in the northwest of Yulin City, mainly concentrated in Shenmu City, Yuyang District, northern Dingbian County and northern Jingbian County. Combined with the spatial expansion of urban construction land in each period, it can be concluded that the spatial degradation of habitat quality in Yulin City is closely related to the expansion of construction land. On the one hand, the expansion of urban construction land has occupied a large amount of ecological space and increased the intensity of land use; On the other hand, northwest Yulin is rich in mineral resources. Due to intensive human activities and intervention, the influence distance of threatening factors has been increased, resulting in a decline in regional habitat quality (Fig. 8).

**Figure 9:**
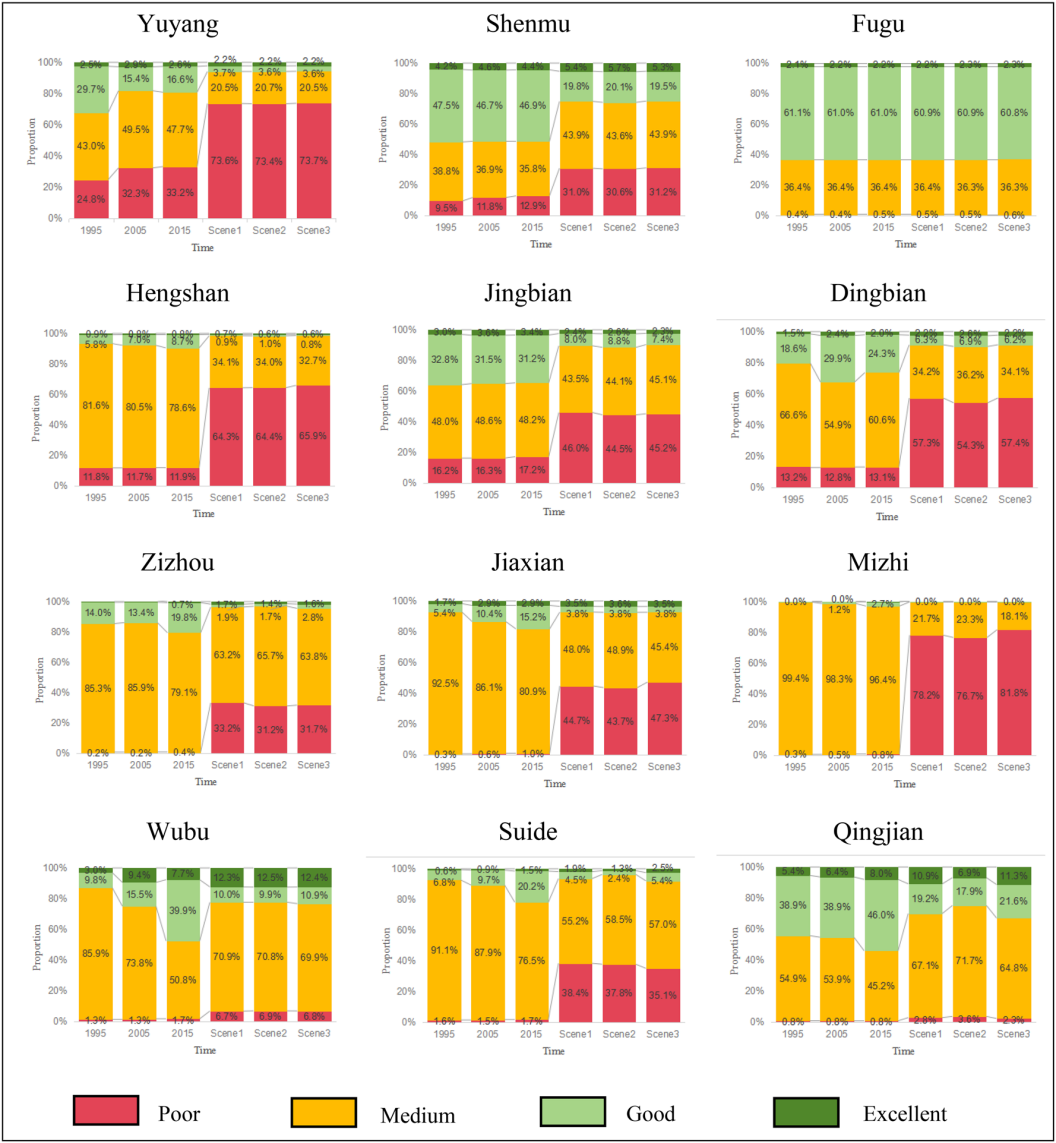
Histogram of habitat quality area ratio in each district of Yulin city.

According to the proportion of habitat quality area in various districts and counties in Yulin City from 1995 to 2015 (Fig. 9), the degree of habitat quality degradation in Yulin City shows the spatial characteristics of “gradually decreasing from west to east”. The slightly degraded areas are Fugu County, Mizhi County and Jia County, the moderately degraded areas are Hengshan District and Zizhou County, and the severely degraded areas are Yuyang District, Shenmu City and Jingbian County.

As the most degraded area in Yulin City in the past 20 years, the area of poor habitat quality in Yuyang District increased by 8.4% (572.05 km^2^), the area of medium ecological quality increased by 4.7% (318.59 km^2^), and the area of good habitat quality decreased by 13.1% (890.64 km^2^). During the study period, Yuyang District vigorously developed urban construction and expanded urban economic development zones on a large scale. The large-scale expansion of built-up areas destroyed the balance of the regional ecosystem and resulted in a significant decline in regional habitat quality. The area of areas with poor habitat quality in Shenmu City and Jingbian County increased by 3.4% (248.01 km^2^) and 1% (47.39 km^2^) respectively, and the areas with good habitat quality decreased by 0.6% (44.33 km^2^) and 1.6% (81.63 km^2^) respectively. The main reason is that the large-scale urban expansion has led to the deterioration of the habitat quality in the area.

The areas with moderate habitat quality degradation in Yulin City are located in Hengshan District and Zizhou County in central Yulin. The poor habitat quality increased by 5.33 and 3.59 km^2^ respectively, and the excellent habitat quality in Hengshan District decreased by 3.77 km^2^. Spatial degradation mainly exists in the forest and grassland in the southeast of Hengshan District and the west of Zizhou County. Due to the impact of artificial surface expansion, ecological services have been reduced and habitat quality has been moderately degraded. During the study period, the habitat quality of Fugu County, Jiaxian County and Mizhi County in the eastern region of Yulin City declined slightly. Because the eastern region of Yulin is close to the Yellow River Basin and has better natural conditions and policy protection, therefore, during the study period, the ecological environment of the region was less affected by human factors, and the habitat quality was also less affected.

#### Comparative analysis of multi-scenario simulation of habitat quality

**Figure 10:**
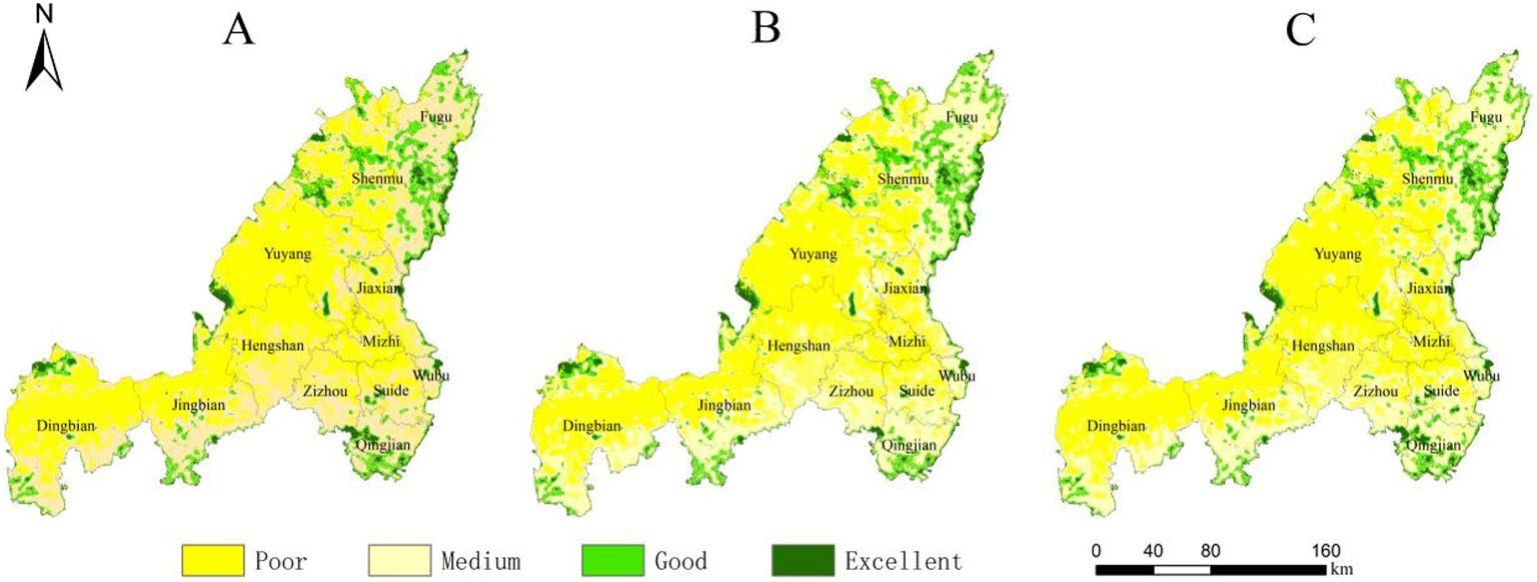
Spatial distribution of Yulin’s habitat quality in 2035 under the scenarios of natural development (**A**), ecological protection (**B**) and suitable development of urban economy (**C**).The maps were created using the free and open source ArcGIS, Version 10.5 (https://www1.jjjzfx.cn/gis-jb61d/).

Based on the multi-scenario land use prediction results of Yulin City in 2035 obtained by the PLUS model, the threat source data within each scenario was extracted and imported into the InVEST model. The results are shown in Fig. 10.

##### Scenario 1

Under the natural development scenario, the habitat quality of the entire Yulin area showed overall degradation (Fig. 10A). Compared with the habitat quality in 2015, the proportion of areas with poor habitat quality under this scenario increased from 13.2% to 46.1%, an increase of approximately 32.9% (14127.36 km^2^). The area proportions of medium and good habitat quality decreased by 13.6% (5799.37 km^2^) and 19.6% (8431.06 km^2^) respectively, while the excellent habitat quality increased by 0.3% (103.07 km^2^) (Fig. 9). Good and excellent habitat quality is mainly distributed in the Hongjiannao area of Shenmu City, the area along the Yellow River in the east of Yulin City, the northwest part of Hengshan District, and the Baiyu Mountains area in the south of Dingbian County and Jingbian County. The habitat quality of the remaining areas is moderately poor, indicating that under the natural development scenario, the habitat quality of Yulin City will face the risk of large-scale degradation in the future.

##### Scenario 2

Under the ecological protection scenario, the proportion of areas with poor habitat quality in Yulin City increased from 13.2% in 2015 to 44.6%, an increase of approximately 31.4% (13500.51 km^2^). The area proportions of medium and good habitat quality decreased by 12.8% (5459.27 km^2^) and 18.9% (8170.59 km^2^) respectively, and the excellent habitat quality increased by 0.4% (129.35 km^2^) (Figs. 9, 10B). Habitat quality degradation is mainly concentrated in the central and western regions of Yulin City. The most obvious degradation is in Yuyang District, where the degraded area increased by 40.2% (2741.98 km^2^). This is mainly due to the degradation of habitat quality caused by artificial surface expansion. Compared with the natural development scenario, the habitat quality of Yulin City will be greatly improved in 2035 under the ecological protection scenario. It shows that ecological protection is effective in maintaining urban habitat quality, but the expansion of artificial surface has a greater impact on regional habitat quality to a certain extent.

##### Scenario 3

Under the appropriate urban economic development scenario, the overall habitat quality of Yulin City has been significantly improved compared to the habitat quality under the natural development scenario (Figs. 9, 10C). Compared with 2015, the area of excellent habitat quality increased by 0.4% (137.09 km^2^), the proportion of areas with medium and good habitat quality decreased by 13.4% (5757.96 km^2^) and 19.1% (8207.25 km^2^) respectively, and the area of poor habitat quality an increase of 32.1% (13828.12 km^2^). The severely degraded areas of habitat quality are located in the expansion areas of construction land in each district, which is enough to show that the expansion of construction land has a high degree of impact on habitat quality.

Through multi-scenario prediction and comparative analysis of habitat quality, under the natural development scenario, artificial surface will maintain the historical expansion trend, and Yulin City may face the risk of large-scale habitat quality degradation by 2035; Under the ecological protection scenario, the habitat quality in the restricted development area is protected to a certain extent, but the expansion of artificial surface will affect the habitat quality in the protected area; Under the scenario of suitable urban economic development, Yulin City’s habitat quality has been improved to a certain extent, which not only protects ecological security but also meets the demand for construction land for urban development. Therefore, in order to improve the habitat quality of Yulin City, the government must not only strictly implement ecological protection boundaries, but also formulate reasonable policies in urban development, control urban scale and development boundaries, optimize ecological compensation mechanisms, improve regional landscape connectivity, and implement ecological restoration strategies, Strengthen the comprehensive improvement of Yulin City’s ecological space.

## Discussion

Based on the land use data of the third period of Yulin City, the PLUS model was used to simulate the land use changes in 2015, and the kappa coefficient was used to test the accuracy of the simulated data and the real data to support the reliability of the research. By setting development zone boundaries and adjusting parameters to design three progressive scenarios, the spatial distribution of land use in Yulin City in 2035 is predicted, and the InVEST model was used to analyze the spatiotemporal evolution of Yulin City’s habitat quality in the past 20 years and evaluate the distribution of Yulin City’s habitat quality under three scenarios after 20 years. This is different from the classic land use scenario simulation^43,44^. Three progressive scenarios are designed to better reflect the degree of impact on habitat quality in scenario design. We found that compared to Scenario 1, the additional boundary restrictions in Scenario 2 have a limited impact on improving regional habitat quality, because human activities outside restricted areas have radiative effects on interior boundary habitat quality, further consideration of conservation spaces based on boundary restrictions will be necessary in the future. In Scenario 3, setting the conversion parameters of construction land according to policy planning and combining with ecological protection restriction areas can effectively improve regional habitat quality, indicating that policy factors play an important role in ecological protection and appropriate urban development. This study helps the government better understand the evolution of land use and habitat quality in Yulin City in the past 20 years, providing theoretical support and reference basis for the formulation of Yulin City’s ecological environment protection policies and the implementation of ecological protection work under the current land and space planning. However, this study still has shortcomings, such as the selection of threat factors in habitat quality is not comprehensive enough. Future studies could explore additional variables or models to capture the full complexity of habitat quality changes. Integrating socio-economic models with ecological models could offer a more holistic view of the interactions between human activities and ecological health.

## Recommendations and conclusions

### Recommendations

**Figure 11:**
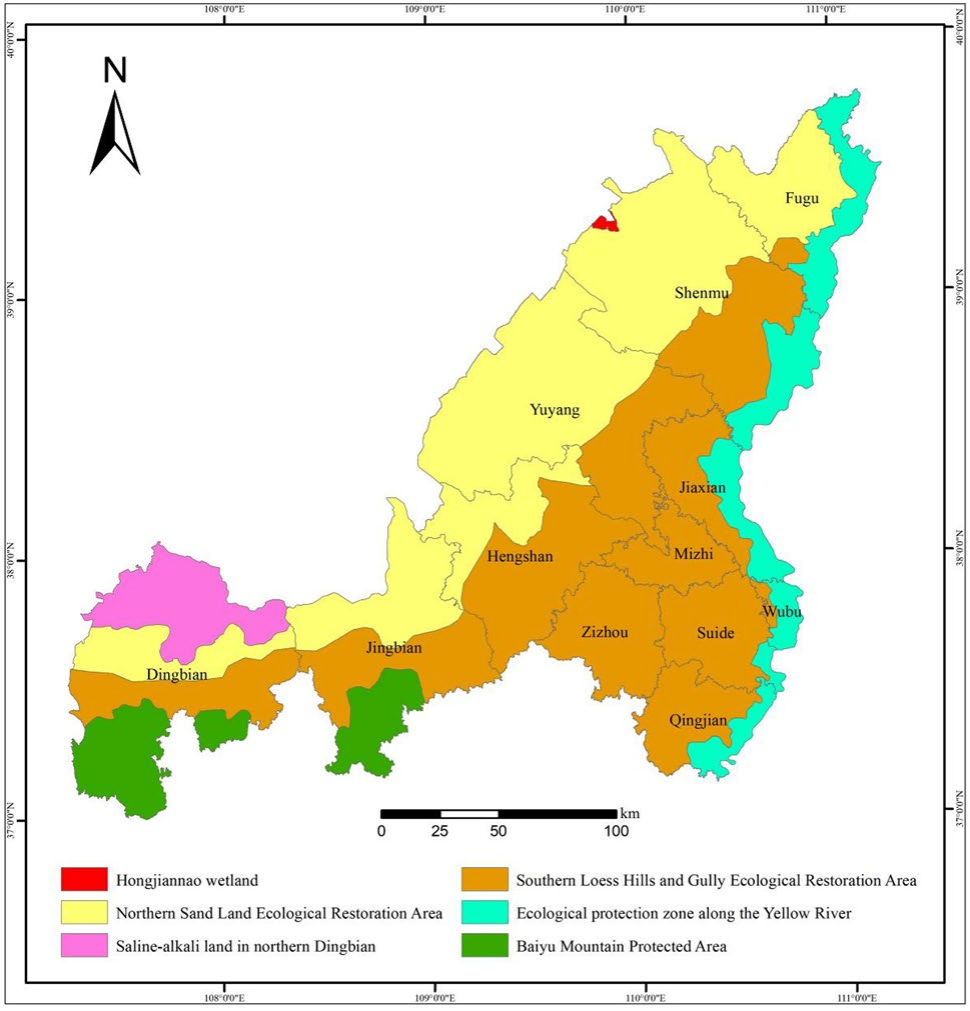
Ecological restoration pattern of Yulin city. The maps were created using the free and open source ArcGIS, Version 10.5 (https://www1.jjjzfx.cn/gis-jb61d/).

According to the policy planning of the “National Important Ecosystem Protection and Restoration Major Projects Master Plan (2021–2035)” and the “Shaanxi Province Land Space Ecological Restoration Plan (2021–2035)”, Yulin City is an important part of the national “two screens and three belts” ecological security pattern, and is also a key national soil and water loss prevention and control area. Based on the above scenario simulation results of future land use changes and habitat quality in Yulin City, combined with relevant policy planning, the following suggestions are put forward to improve the quality of the ecosystem in Yulin City:

- (1) Strengthen the connection with provincial planning, and build an ecological restoration pattern of Yulin City’s “One Belt, Two Districts and Three Points” based on the habitat quality classification results and topography classification (Fig. 11). That is, the ecological protection zone along the Yellow River, northern sand land ecological restoration area, southern loess hilly and gully ecological restoration area, the saline-alkali land in the north of Jingbian, the Baiyu mountain protection area, and the Hongjiannao Wetland.
- (2) The ecological protection zone along the Yellow River involves 6 counties: Fugu County, Shenmu City, Jiaxian County, Wubao County, Suide County and Qingjian County, with an area of 3743.76 km^2^. The habitat quality in this area is good, and the quality of the ecosystem can be improved through protection and conservation, by establishing ecological reserves along the Yellow River, removing stress factors, building ecological corridors, in-situ and ex-situ protection, and breeding rare and endangered biological species. Protect ecosystem integrity, protect biodiversity, and improve ecosystem quality.
- (3) For the northern sand land ecological restoration area, a total of 6 counties including Fugu County, Shenmu City, Yuyang District, Hengshan District, Jingbian County and Dingbian County are involved, covering an area of 16151.51 km^2^. The quality of the habitat in this area is poor, and it is necessary to improve the quality of the ecosystem through ecological reconstruction and assisted regeneration. On the basis of eliminating stress factors, measures should be taken to improve the physical environment, introduce suitable species, and focus on reshaping the landscape, reconstructing the habitat, restoring vegetation, and biological Carry out ecological reconstruction in terms of diversity reorganization and other aspects.
- (4) The southern loess hilly gully ecological restoration area involves 12 counties and districts including Zizhou County, Suide County, and Mizhi County, with a total area of 18175.91 km^2^. The overall habitat quality of this area is medium, and the comprehensive restoration of the mountains, rivers, forests, fields, lakes and grass systems can be carried out following the principle of giving priority to natural restoration and supplementing it with artificial restoration. Restore degraded vegetation and cultivated land, and build horizontal ditches and fish scale pits in the Liangmao area to prevent water and soil erosion, thereby improving the region's water conservation capacity and the quality of the ecosystem.
- (5) For the saline-alkali land in the north of Dingbian, which covers an area of 2056.76 km^2^, we can implement saline-alkali land ecological restoration projects, carry out deep plowing and plowing, increase the application of organic fertilizers and other measures to alleviate soil salinization and gradually reduce soil salinity; Promote the introduction and cultivation of saline-alkali tolerant plant species to promote the succession of saline-alkali land to grassland, thereby improving the quality of the ecosystem.
- (6) For the Baiyushan protected area and Hongjiannao Wetland, with an area of 2824.2 km^2^ and 47.71 km^2^ respectively, protected areas can be established to expand the living space of wild animals and plants in the protected areas and improve biodiversity in the protected areas; restoration is guided by water source conservation and biodiversity protection to ensure the stability of ecosystem service functions in the protected area.

## Conclusions

- (1) During the study period, construction land in Yulin City expanded rapidly and ecological land gradually shrank. Among them, cultivated land, grassland and water area have been transferred to construction land on a large scale. In 20 years, the construction land area has increased by 380.87km^2^, accounting for about 245% of the original construction land area. The urban spatial expansion of Yulin is mainly based on external expansion, which meets the needs of urban development by encroaching on external ecological space.
- (2) By comparing the land use changes of three scenarios in Yulin City in 2035, it can be concluded that land use changes are mainly concentrated in the central and western regions. Under the natural development scenario, since there are no boundary constraints, construction land encroaches on ecological land on a large scale, causing serious damage to the urban ecosystem. Under the ecological protection scenario, the area of ecological land such as woodlands, grasslands, and waters has increased, and boundary constraints play an important role in ecological protection and the optimization of urban ecosystems. Under the scenario of suitable urban economic development, not only the ecological land space can be protected, but also the demand for construction land in future urban development planning can be met.
- (3) During the study period, the habitat quality of Yulin City was at a medium level from the classification level, and the overall habitat quality showed a downward trend. The area of poor habitat quality increased by 968.94 km^2^. From the perspective of spatial distribution, the severely degraded areas of habitat quality are mainly located in the northwest of Yulin City, and the degree of habitat quality degradation in Yulin City shows the spatial characteristics of “gradually decreasing from west to east.”
- (4) Through a comparative analysis of the habitat quality of Yulin City under three scenarios in 2035, we draw the following conclusions: Under the natural development scenario, the habitat quality of Yulin City faces the risk of widespread degradation in 2035. Under the ecological protection scenario, the habitat quality in restricted development areas is protected to a certain extent, but the expansion of artificial surfaces will also affect the habitat quality in protected areas. Under the scenario of suitable urban economic development, Yulin City’s habitat quality has been improved to a certain extent, which not only protects ecological security but also meets the demand for construction land for urban development.

## Data Availability

The three-phase land use data required for this study comes from the Resource and Environmental Science Data Center of the Chinese Academy of Sciences (https://www.resdc.cn/Datalist1.aspx?FieldTyepID=1,3). The DEM data comes from the geospatial data cloud (https://www.gscloud.cn/), the slope is extracted from the DEM data, and the soil type, temperature, precipitation, population and GDP data come from the Resource and Environment Data Sharing Center of the Chinese Academy of Sciences (https://www.resdc.cn/). Data such as water systems, roads and administrative centers come from the National Geographic Information Resources Directory Service System (https://www.webmap.cn/main.do?method=index). I ensure that the data availability statement given on the system under Declarations and given in the Manuscript are same. Data are available upon reasonable request, please contact the corresponding author.

## References

[1] Hall LS, Krausman PR, Morrison ML (1997). The habitat concept and a plea for standard technology. Wildl. Soc. Bull..

[2] Liu C, Wang C (2018). Spatial and temporal evolutionary characteristics of habitat quality in loess hilly areas based on land use change—An example from Yuzhong county. J. Ecol..

[3] Feng S, Sun R, Chen L (2018). Spatial and temporal evolution of habitat quality in Beijing based on land use pattern changes. J. Ecol..

[4] Han R, Zhang J, Zhu W, Wang L, Zhang L, Zhu L (2018). Impact of land use change on habitat in the Qihe river basin of Taihang mountains. Prog. Geogr..

[5] Zhang X, Zhou J, Li M (2020). Analysis on spatial and temporal changes of regional habitat quality based on the spatial pattern reconstruction of land use. Acta Geogr. Sin..

[6] Feng S, Sun RH, Chen LD (2022). Spatio-temporal characteristics of habitat quality based on land-use changes in Guangdong province. Acta Ecol. Sin..

[7] Chu L, Sun T, Wang T, Li Z, Cai C (2018). Evolution and prediction of landscape pattern and habitat quality based on CA-Markov and InVEST model in Hubei section of three gorges reservoir area (TGRA). Sustainability.

[8] Sang L, Zhang C, Yang J, Zhu D, Yun W (2011). Simulation of land use spatial pattern of towns and villages based on CA-Markov model. Math. Comput. Model..

[9] Chu L, Zhang XR, Wang TW, Zhao X (2018). Spatial-temporal evolution and prediction of urban landscape pattern and habitat quality based on CA-Markov and InVEST model. J. Appl. Ecol..

[10] Wang G, Yang K, Xu Q, Liu T, Wang B (2015). Model of dianchi basin land use change based on CA and MAS. Environ. Sci. Technol..

[11] Verburg P, Soepboer W, Veldkamp A, Limpiada R, Espaldon V, Mastura S (2002). Modeling the spatial dynamics of regional land use: The CLUE-S model. Environ. Manag..

[12] Shahidul I, Yuechen L, Mingguo M, Anxu C, Zhongxi G (2021). Simulation and prediction of the spatial dynamics of land use changes modelling through CLUE-S in the Southeastern region of Bangladesh. J. Indian Soc. Remote Sens..

[13] Artikanur SD, Widiatmaka W, Setiawan Y, Marimin M (2022). Predicting sugar balance as the impact of land-use/land-cover change dynamics in a sugarcane producing regency in East java. Indones. Front. Environ. Sci..

[14] Liu X, Liang X, Li X, Xu X, Ou J, Chen Y, Li S, Wang S, Pei F (2017). A future land use simulation model (FLUS) for simulating multiple land use scenarios by coupling human and natural effects. Landsc. Urb. Plan.

[15] Yingxue L, Zhaoshun L, Shujie L, Xiang L (2022). Multi-scenario simulation analysis of land use and carbon storage changes in Changchun city based on FLUS and InVEST model. Land.

[16] Liang X, Liu X, Li X, Chen Y, Tian H, Yao Y (2018). Delineating multi-scenario urban growth boundaries with a CA-based FLUS model and morphological method. Landsc. Urb. Plan..

[17] Li S, Hong Z, Xue X, Zhang F, Shi W (2022). Multi-scenario simulation of LUCC in Binzhou city based on logistic-CA-Markov coupling model. Res. Soil Water Conserv..

[18] She J, Guan Z, Cai F, Pu L, Tan J, Chen T (2017). Simulation of land use changes in a coastal reclaimed area with dynamic shorelines. Sustainability.

[19] Qiao Z, Jiang Y, He T, Lu Y, Xu X, Yang J (2022). Advances in land-use change simulation research. J. Ecol..

[20] Haiyan H, BingBing Z, Fengsong P, Guohua H, Zhongbo S, Yijian Z, Han Z, Yukun G, Meng L, Xia L (2022). Future land use/land cover change has nontrivial and potentially dominant impact on global gross primary productivity. Earth’s Future.

[21] Liang X, Guan Q, Clarke KC, Liu S, Wang B, Yao Y (2021). Understanding the drivers of sustainable land expansion using a patch-generating land use simulation (PLUS) model: A case study in wuhan. Ch. Comput. Environ. Urb. Syst..

[22] Lai Z, Chen C, Chen J, Wu Z, Wang F, Li S (2022). Multi-scenario simulation of land-use change and delineation of urban growth boundaries in county area: A case study of Xinxing county Guangdong province. Land.

[23] Xu L, Guo C, Luo S (2023). Study on habitat quality in Kunming based on PLUS model and InVEST model. Ecol. Environ. Monit..

[24] Yang S, Su H, Zhao G (2022). A multi-scenario simulation of urban ecosystem service value based on PLUS model—Hanzhong city as an example. Arid. Zone Resour. Environ..

[25] Wang J, Wang W, Hai M (2022). Simulation analysis of land use change in Shandong province based on PLUS model. Land Nat. Resour. Res..

[26] Villa, F., Ceroni, M., Bagstad, K., Gary, J. & Sergey, L. “ARIES (artificial intelligence for ecosystem services): A new tool for ecosystem services assessment, planning, and valuation,”. In *Proc. 11th Annual BioEcon Conference on Economic Instruments to Enhance the Conservation and Sustainable Use of Biodiversity* (2009).

[27] Brown G, Brabyn L (2012). The extrapolation of social landscape values to a national level in New Zealand using landscape character classification. Appl. Geogr..

[28] Liu M, Zhang H, Wang Y, Pei H (2021). Characteristics of habitat quality in the agro-pastoral ecotone of Northern China based on land uses. Res. Soil Water Conserv..

[29] Salata S, Garnero G, Barbieri CA, Giaimo C (2017). The integration of ecosystem services in planning: An evaluation of the nutrient retention model using InVEST software. Land.

[30] Ding Y, Wang L, Gui F, Zhao S, Zhu FW (2023). Carbon stocks in the circum Hangzhou bay ecosystem based on the InVEST model and PLUS model. Environ. Sci..

[31] Wu L, Sun C, Fan F (2021). Estimating the characteristic spatiotemporal variation in habitat quality using the InVEST model—A case study from Guangdong-HongKong-Macao greater bay area. Remote Sens..

[32] Liu H, Lin M, Zhou R, Zhong L (2021). Spatial-temporal evolution analysis of habitat quality in Guangdong-Hong Kong-Macao greater bay area based on InVEST model. Ecol. Sci..

[33] Jing X, Zhao Q (2021). Research on the spatiotemporal changes of habitat quality in Guizhou province based on the InVEST model. Territ. Nat. Resour. Study.

[34] Liang X, Yuan L, Ning L, Song C, Cheng C, Wang X (2020). Spatial pattern of habitat quality in Heilongjiang province and its influencing factors based on InVEST model. J. Beijing Normal Univ. Sci..

[35] Feng W, Lin M, Gong J, Zhao J, Zhong L, Liu H (2022). Temporal and spatial differentiation characteristics of habitat quality in Zhongshan city based on FLUS-In VEST model. Ecol. Sci..

[36] Gao Z, Wang X, Sui X, Wang X, Fan Y, Zhu Q (2022). Multi-scenario prediction of habitat quality in nanjing based on FLUS and InVEST models. J. Agric. Resour. Environ..

[37] Sun X, Xue J, Dong L (2023). Temporal and spatial change and prediction of carbon storage in nanjing ecosystem based on PLUS model and InVEST model. Ecol. R. Environ..

[38] Zhang S, Yang P, Xia J, Wang W, Cai W, Chen N (2022). Land use/land cover prediction and analysis of the middle reaches of the Yangtze river under different scenarios. Sci. Total Environ..

[39] Wang J, Wu Y, Gou A (2023). Habitat quality evolution characteristics and multi-scenario prediction in Shenzhen based on PLUS and InVEST models. Front. Environ. Sci..

[40] Wang Y, Lan A, Fan Z, Lin S, Zhu N (2023). Analysis of spatial and temporal variation characteristics and drivers of habitat quality in the Chishui river basin based on the InVEST model. Ch. R. Water Hydropow..

[41] Zhao Y, Qu Z, Zhang Y, Ao Y, Han L, Kang S (2022). Effects of human activity intensity on habitat quality based on nighttime light remote sensing: A case study of Northern Shaanxi. Ch. Sci. Total Environ..

[42] Lei J, Chen Y, Chen Z, Chen X, Wu T, Li Y (2022). Spatial and temporal evolution of habitat quality in three watersheds of Hainan Island based on the InVEST model. J. Appl. Ecol..

[43] Yang L, Yang J, Zhou W (2023). Coupling evolution analysis of LUCC and habitat quality in Dongting lake basin based on multi-scenario simulation. Ch. Environ. Sci..

[44] Chen L, Cai H, Zhang T, Zhang X, Zeng H (2022). Multi-scenario simulation analysis of land use in Rao river basin based on Markov-FLUS model. Acta Ecol. Sin..

